# Neurobiological mechanisms of olfactory dysfunction: a ten-year bibliometric and visualization analysis

**DOI:** 10.3389/fmed.2026.1812327

**Published:** 2026-05-13

**Authors:** Yuxin Zhu, Yutian Chen, Genki Izumoji, Siying Qu, Donghai Wu, Huiqin Chu, Yixiang Wang, Haiju Sun, Xiaoyu Li

**Affiliations:** 1 The Third Affiliated Hospital of Zhejiang Chinese Medical University (Zhongshan Hospital of Zhejiang Province), Hangzhou, Zhejiang, China; 2 Key Laboratory of Acupuncture and Neurology of Zhejiang Province, The Third Clinical Medical College, Zhejiang Chinese Medical University, Hangzhou, Zhejiang, China; 3 The First Affiliated Hospital of Zhejiang Chinese Medical University (Zhejiang Provincial Hospital of Traditional Chinese Medicine), Hangzhou, Zhejiang, China

**Keywords:** bibliometric analysis, CiteSpace, mechanisms, olfactory dysfunction, VOSviewer

## Abstract

**Background:**

Olfactory dysfunction (OD) has gained prominence in neurodegenerative diseases and COVID-19 sequelae in recent years. Its mechanisms have also attracted increasing research interest. However, there is currently a scarcity of bibliometric analyses in this field.

**Methods:**

Articles related to OD mechanisms were searched in the Web of Science Core Collection (WoSCC) and Scopus. Data merging and bibliometric analysis were conducted using CiteSpace, VOSviewer, Excel, Scimago Graphica, and the bibliometrix in R package. Simultaneously, PubMed was used to search and summarize interventional clinical trials in this field, and their protocols were tracked through trial registration information and ethical approval records.

**Results:**

A total of 7,915 articles met the inclusion criteria in WoSCC and Scopus. Overall, the number of articles published annually on the mechanisms of OD is on the rise. The USA (2635 publications), University of California System (143 publications), and Thomas Hummel (174 publications) are the most productive country, institution, and author, respectively. Keyword analysis shows that “COVID-19,” “Parkinson’s disease,” “inflammation,” “odorant receptor,” and other related topics are hot topics and trends in research. PubMed retrieved and included 14 interventional clinical trials. These trials mainly focus on pharmacological interventions, non-pharmacological interventions, surgical interventions, and mechanistic studies.

**Conclusion:**

Mechanistic research on OD is advancing from macroscopic observations to precise molecular mechanisms. This review synthesizes evidence on how distinct etiologies, ranging from post-viral and inflammatory damage to neurodegeneration and metabolic imbalances, contribute to OD. Notably, the dysregulation of the inflammatory NF-κB, signal-transducing cAMP, and neuroregenerative Wnt/β-catenin pathways may collectively contribute to the development of OD. By integrating bibliometric trends with clinical trial evaluations, this study delineates a clear translational pipeline from mechanism exploration to clinical interventions.

## 1 Introduction

Olfactory dysfunction (OD) is a prevalent sensory condition primarily classified into quantitative disorders (hyposmia, anosmia, hyperosmia) and qualitative disorders (parosmia, phantosmia) (1), psychophysically characterized by elevated odor detection thresholds and compromised discrimination ability (2). As an increasingly common and pathological condition, the prevalence of OD increases with age, and its incidence in the general population is approximately 22%, exhibiting highly significant epidemiological characteristics and substantial clinical warning value (3, 4). Beyond isolated sensory deficits, OD is closely linked to a broad spectrum of neurological and systemic disorders. It has been reported in up to 90% of patients with early-stage Parkinson’s disease (PD) and 85% of patients with early Alzheimer’s disease (AD), suggesting its potential as a non-invasive clinical biomarker for the early screening of neurodegenerative diseases (5). Furthermore, OD is a frequent symptom of SARS-CoV-2 infection, even in the absence of overt nasal manifestations such as congestion or rhinorrhea, data indicate that 79.7% of patients without nasal symptoms still report olfactory abnormalities (6). Emerging evidence also supports associations with cardiovascular diseases, nutritional deficiencies, and immunological disorders (7). Given its high prevalence and subtle onset, mechanistic research is vital for early diagnosis and public health management.

Despite growing interest, the pathophysiology of OD remains incompletely elucidated. The current evidence chain exhibits a hierarchical characteristic ranging from the macroscopic microenvironment to the microscopic molecular regulation. Existing studies have shown that local and systemic inflammation play a significant pathogenic driving role: inflammatory cytokines not only directly disrupt the homeostasis of the olfactory epithelium (OE) or olfactory bulb (OB) (8, 9), but this continuous inflammatory stress also further inhibits the neuroregenerative capacity at the cellular level. Unlike most peripheral neurons, olfactory sensory neurons (OSNs) have the capacity for continuous renewal; however, this periodic repair process can be blocked under inflammatory conditions, resulting in damaged neurons not being replaced in time (10, 11). More in-depth molecular-level research has revealed that even if some neurons survive, dysregulation of olfactory receptors (ORs) expression and the blockade of downstream signal cascades can still lead to further impairment of olfactory function (12). Therefore, cross-level integration and strengthening etiological links are likely effective strategies for early OD diagnosis and clinical translation.

Bibliometric analysis provides quantitative tools to characterize publication trends, citation patterns, and collaborative networks, yielding a holistic overview of a research field (13). However, existing reviews on OD mechanisms often suffer from limitations such as focusing on individual subfields (5), oversimplifying mechanism explanations (14), and presenting asymmetrical evidence for peripheral and central mechanisms (15). Such deficiencies may obscure the intrinsic connections between different pathological processes and potentially impede the translation of fundamental discoveries into clinical practice. To fill this gap, this study systematically maps the knowledge landscape of OD mechanism research over the past decade, by innovatively integrating bibliometric analysis with clinical trial analysis. Utilizing advanced analytical tools (CiteSpace, VOSviewer, and the bibliometrix in R package) (16, 17), we analyzed publication trends, collaboration networks, and keyword evolution. On this basis, informed by the macro-level themes identified through bibliometric clustering, highly cited core literature and recurring process-level themes observed across different disease contexts, we conducted a targeted manual review to qualitatively synthesize etiology-specific neurobiological mechanisms and representative signaling pathways, including inflammatory signaling, neuroregenerative impairment, and olfactory signal transduction. This integrated approach was intended to connect bibliometric trends with literature-based biological interpretation, thereby highlighting emerging research frontiers and unmet needs in the field and providing a basis for future mechanistic and translational research on OD.

## 2 Materials and methods

### 2.1 Design and data acquisition

**FIGURE 1:**
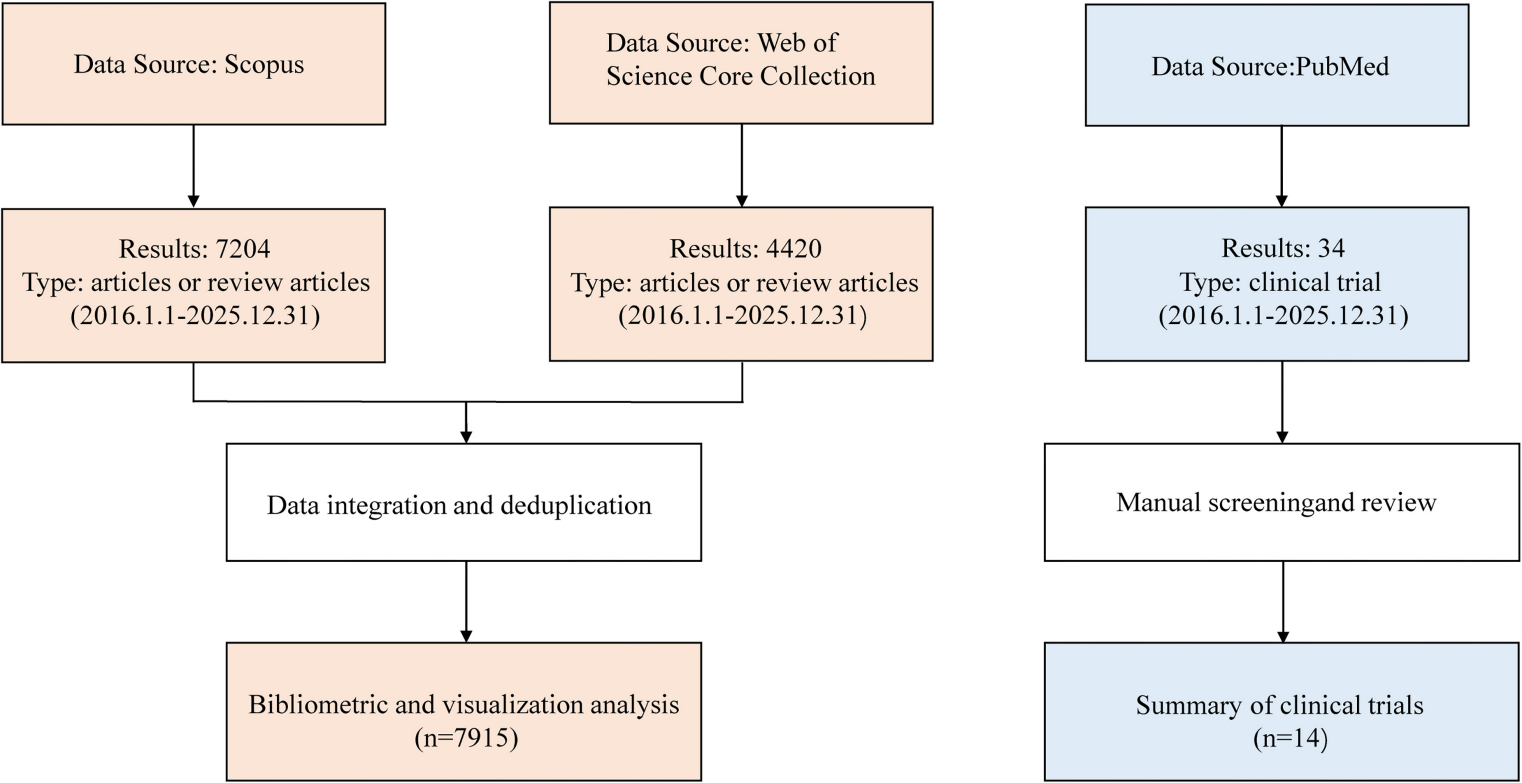
Flowchart of data collection and study design.

The data for this study were sourced from the Web of Science Core Collection (WoSCC), Scopus, and the PubMed database (Figure 1 and Table 1). We narrowed down the search scope to the period from January 1, 2016, to December 31, 2025. To ensure the academic rigor of the dataset, strict inclusion and exclusion criteria were applied in WoSCC and Scopus: (1) only peer-reviewed “Articles” and “Review Articles” in English were included; (2) conference papers, news reports, editorials, early access articles, and records lacking core metadata or abstracts were excluded. Bibliographic data were then downloaded in plain text and CSV formats, including full records and cited references. Python (version 3.11) was used both to convert Scopus CSV files into a unified plain text format consistent with the WoSCC full record and cited references structure and to conduct data cleaning, including DOI-based deduplication; removal of retracted publications; exclusion of records with “[Anonymous]” in the author field; and other standardization procedures. The final validated dataset comprised 7,915 publications for downstream bibliometric analysis. Meanwhile, in PubMed, we initially retrieved 3,693 articles. A targeted parallel retrieval was also conducted in PubMed to supplement the clinical translational evidence related to the mechanism studies. After applying the “Clinical Trial” article type, a total of 14 interventional clinical trials were obtained through manual screening and review.

**TABLE 1:** Search strategy via Web of Science Core Collection, Scopus, and PubMed.

Step	Web of Science Core Collection strategy	PubMed strategy	Scopus strategy
#1	TS = (“olfactory dysfunction” OR “olfactory disorder*” OR “anosmia” OR “hyposmia” OR “dysosmia” OR “parosmia” OR “olfactory impair*” OR “olfactory loss” OR “olfactory deficit*” OR “olfaction disorder*” OR “smell loss” OR “smell disorder*” OR “smell impair*” OR “smell deficit*” OR “smell” OR “olfaction”)	“olfactory dysfunction”[Title/Abstract] OR “olfactorydisorder*”[Title/Abstract] OR “anosmia”[Title/Abstract] OR “hyposmia”[Title/Abstract] OR “dysosmia”[Title/Abstract] OR “parosmia”[Title/Abstract] OR “olfactory impair*”[Title/Abstract] OR “olfactory loss”[Title/Abstract] OR “olfactory deficit*”[Title/Abstract] OR “olfaction disorder*”[Title/Abstract] OR “smell loss”[Title/Abstract] OR “smell disorder*”[Title/Abstract] OR “smell impair*”[Title/Abstract] OR “smell deficit*”[Title/Abstract] OR “smell”[Title/Abstract] OR “olfaction”[Title/Abstract]	TITLE-ABS-KEY(“olfactory dysfunction” OR “olfactory disorder*” OR “anosmia” OR “hyposmia” OR “dysosmia” OR “parosmia” OR “olfactory impair*” OR “olfactory loss” OR “olfactory deficit*” OR “olfaction disorder*” OR “smell loss” OR “smell disorder*” OR “smell impair*” OR “smell deficit*” OR “smell” OR “olfaction”)
#2	TS = (“mechanism*” OR “patholog*” OR “pathophysiolog*” OR “etiolog*” OR “neurobiolog*” OR “neuropatholog*”)	“mechanism*”[Title/Abstract] OR “patholog*”[Title/Abstract] OR “pathophysiolog*”[Title/Abstract] OR “etiolog*”[Title/Abstract] OR “neurobiolog*”[Title/Abstract] OR “neuropatholog*”[Title/Abstract])	TITLE-ABS-KEY(“mechanism*” OR “patholog*” OR “pathophysiolog*” OR “etiolog*” OR “neurobiolog*” OR “neuropatholog*”)
#3	#1 AND #2	#1 AND #2	#1 AND #2

### 2.2 Data analysis and visualization

To reveal the research landscape and development trends in the field of OD mechanisms, this study combined the use of CiteSpace 6.4.R1, VOSviewer 1.6.19, Excel, Scimago Graphica and the bibliometrix in R package. The two most important software packages are CiteSpace and VOSviewer. CiteSpace can visually present the structure, patterns, and distribution of scientific knowledge, with the advantage of focusing on analyzing the potential knowledge contained in scientific research (16). VOSviewer is a program designed for constructing and visualizing bibliometric maps, particularly useful for examining maps that include a moderate to substantial number of items (18).

The records retrieved from WoSCC and Scopus were downloaded and imported into the aforementioned software tools for visualization analysis. We used CiteSpace to identify keyword mutations, journal flow, and national/institutional/author collaboration relationships. The general parameter settings were as follows: the time span was from January 2016 to December 2025; the time slice length was 1 year; the connection strength was selected as Cosine; and the network range was set as Within Slices. The specific parameter settings for each graphic could be found in the corner of the respective figure. VOSviewer was used to construct and visualize the keyword clustering network based on its default layout algorithm, with the minimum keyword occurrence threshold set at 114. We also used Excel to draw an annual publishing trend chart, visually displaying the growth trend of the field. At the same time, Scimago Graphic was used to construct a location-based research collaboration network diagram, where node size is mapped to the total number of documents, node color is mapped to the total number of citations, and node labels are set to country names. Finally, the bibliometrix in R package was used to construct a thematic map based on centrality and density indicators to reveal the evolutionary pattern of the research field. The parameter settings utilized functions such as convert2df and results < - biblioAnalysis (19).

### 2.3 Exploratory clinical evidence synthesis and ethical validation

To bridge the fundamental mechanism studies with clinical translation, we summarized and organized the 14 interventional clinical trials related to OD in PubMed. It should be explicitly stated that this section is intended as an exploratory and descriptive synthesis rather than a formal systematic review, given the currently limited number of available trials (*n* = 14) in this specific niche. We focused on their research subjects, intervention measures, main outcomes, and methodological limitations. For studies with ethical approval information or clinical trial registration numbers, we further verified their ethical approval records, and their protocols were tracked through registration platforms such as ClinicalTrials.gov to ensure the correspondence between research design, implementation process, and result reporting, thereby enhancing the standardization and credibility of data analysis in this study.

## 3 Results

### 3.1 Annual publication trends and growth stages

**FIGURE 2:**
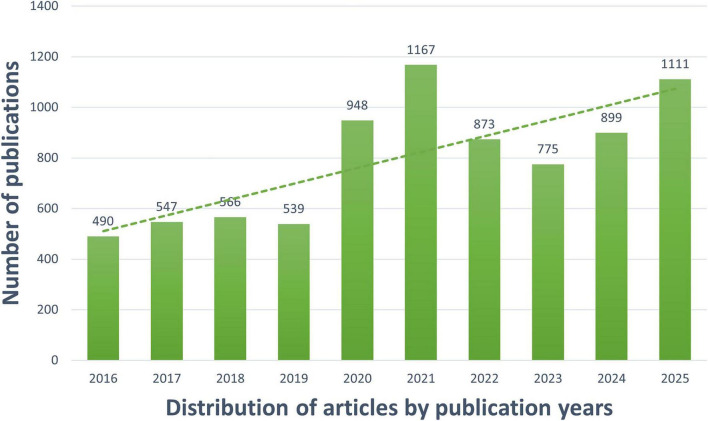
Annual publications from 2016 to 2025.

We searched 7915 articles on the mechanism of OD from 2016 to 2025 in WoSCC and Scopus. By drawing a stacked bar chart (Figure 2), we found that the number of publications on the OD mechanism was relatively low and stable from 2016 to 2019 and showed a rapid upward trend from 2020 to 2021, which may be associated with the outbreak of COVID-19. Since 2022, the quantity has fluctuated. Overall, the number of publications is on the rise, indicating an increasing amount of research on the mechanisms of OD.

### 3.2 Analysis of countries/regions and institutions

As shown in Table 2, the top 10 countries related to this research were identified. In CiteSpace, centrality is a metric that measures the importance of nodes within a network. Observations show that among the top 10 countries, France has the highest centrality (centrality = 0.15), while the USA ranks first in terms of the number of publications (count = 2635). China ranks second in terms of publication volume, but its centrality remains lower than that of several major European countries (count = 1370, centrality = 0.09), suggesting that its role in the international collaboration network could be further strengthened.

**TABLE 2:** The top 10 influential countries/regions.

Rank	Country/region	Count	Centrality
1	USA	2635	0.08
2	China	1370	0.09
3	Germany	722	0.11
4	Italy	583	0.05
5	England	565	0.12
6	Japan	443	0.07
7	France	398	0.15
8	India	335	0.04
9	Canada	305	0.07
10	Spain	294	0.07

**FIGURE 3:**
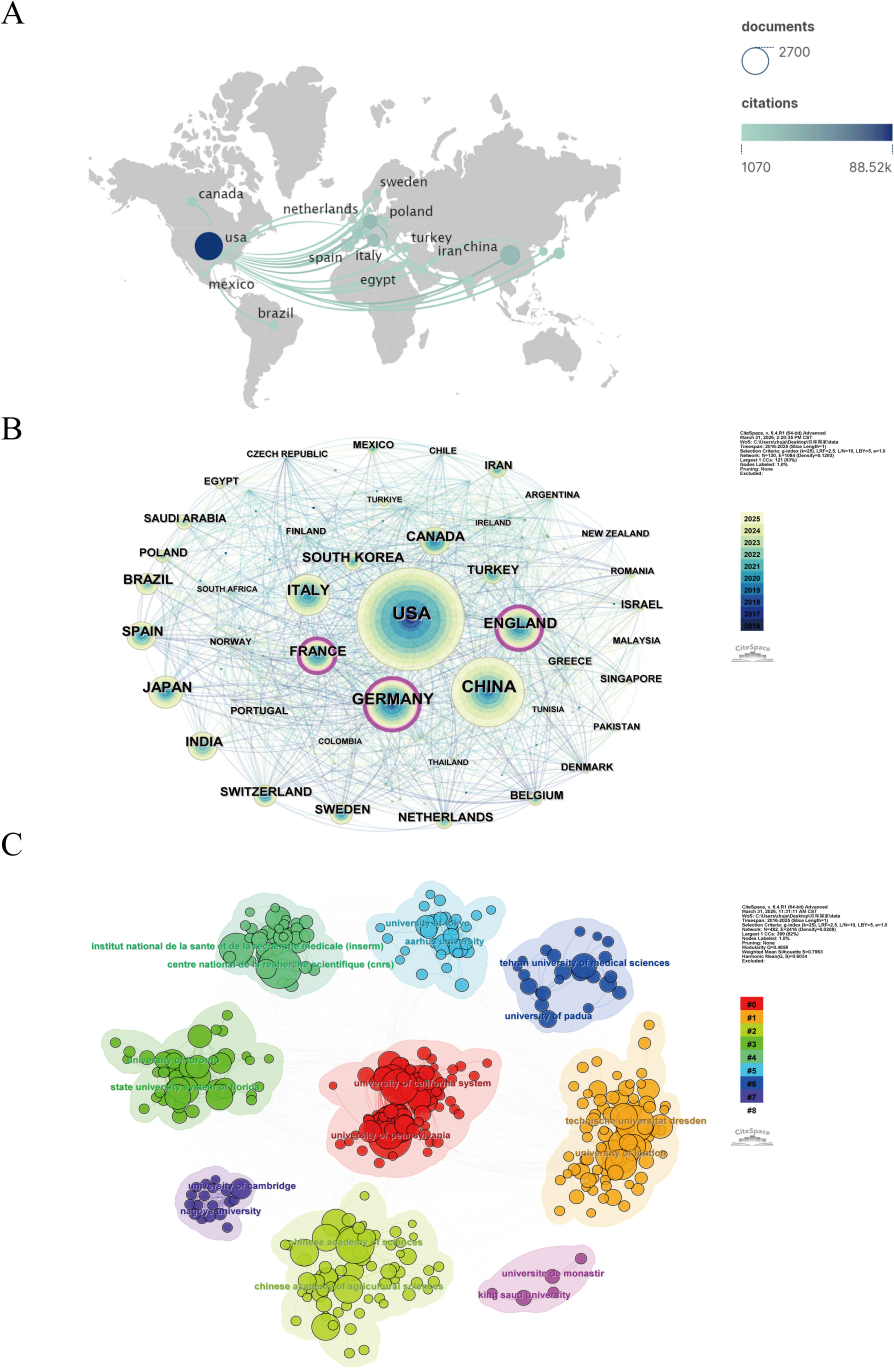
**(A)** Geographical visualization of the international collaboration network and citation impact. The size and color intensity of the circles represent the number of citations for a country’s related scientific research achievements. **(B)** The collaboration network map of countries/regions. The size of the nodes represents the volume of publications by country/region, with larger nodes indicating higher publication volumes; the lines represent collaborative relationships, and denser lines indicate closer collaborations. **(C)** The clustering analysis of the institutional collaboration network. Each node represents an institution. The larger the node, the higher the volume of publications or the frequency of appearance of the institution. Nodes of the same color belong to the same institutional collaboration group.

In the overall citation network (Figure 3A), the legend indicates citation counts ranging from 1,070 to 88,520. The USA has the largest and darkest blue circle, signifying that its research achievements in the field of OD mechanisms are the most frequently cited in the international network, occupying a central position. In Figure 3B, The purple rings outside the nodes indicate higher betweenness centrality, with the purple outer rings of France, England, and Germany being more pronounced, indicating that they not only have high research output but also play an important role in connecting international collaborations.

At the institutional level (Table 3), the University of California System ranked first (count = 143, centrality = 0.05), followed closely by Centre National de la Recherche Scientifique (CNRS) (count = 138, centrality = 0.06). Further examination of the institutional collaboration network (Figure 3C) reveals the formation of multiple relatively independent yet closely connected scientific research clusters in this field. The primary clusters are centered around institutions such as the University of California System, the University of Pennsylvania, the Chinese Academy of Sciences, and the CNRS in France. Overall, institutional groups from the USA, Europe, and China jointly construct a globalized research network.

**TABLE 3:** The top 10 influential institutions.

Rank	Institutions	Count	Centrality
1	University of California System	143	0.05
2	Centre National de la Recherche Scientifique (CNRS)	138	0.06
3	University of Pennsylvania	131	0.04
4	Chinese Academy of Sciences	106	0.09
5	Technische Universitat Dresden	104	0.06
6	Harvard University	96	0.03
7	University of London	95	0.02
8	Institut National de la Sante et de la Recherche Medicale (Inserm)	80	0.02
9	University College London	71	0.02
10	Johns Hopkins University	67	0.01

### 3.3 Analysis of authors

**FIGURE 4:**
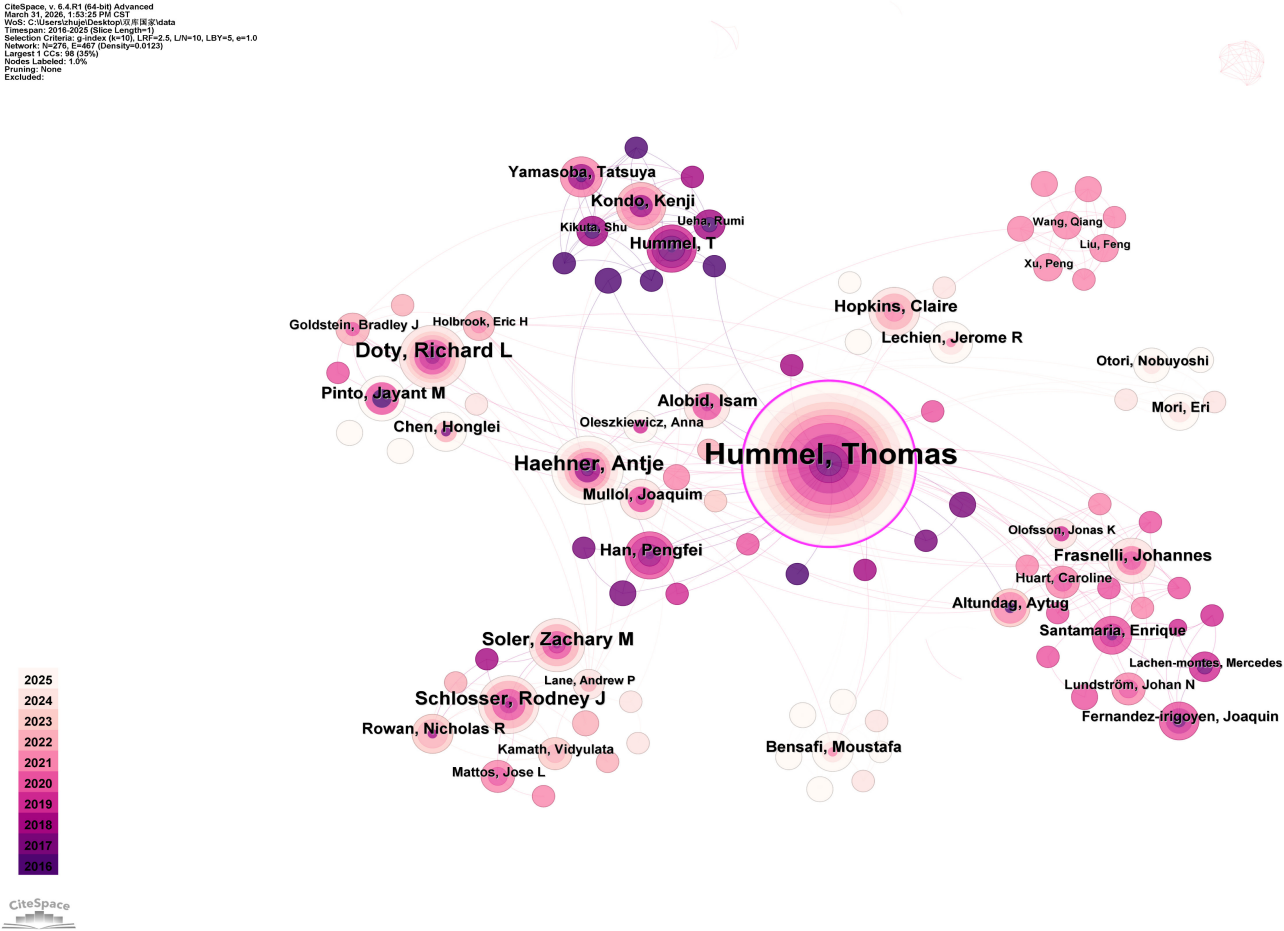
The collaboration network map of authors.

In the author collaboration network (Figure 4), the size of the nodes represents the frequency of the authors’ appearance, while the connections between nodes indicate the collaborative relationships among the authors. The relatively low network density indicates that collaboration in this field is relatively dispersed and that close cooperation among research teams remains limited. As shown in Table 4, Hummel, Thomas (Germany) ranked first, followed by Haehner, Antje (Germany) and Doty, Richard L (USA). Most of the productive authors were affiliated with institutions in Europe and the USA. These authors demonstrated high productivity in the field of OD research, reflecting their important and sustained contributions to this area.

**TABLE 4:** The top 10 most productive authors.

Rank	Author	Affiliation	Country	Count
1	Hummel, Thomas	Technische Universität Dresden	Germany	174
2	Haehner, Antje	Technische Universität Dresden	Germany	37
3	Doty, Richard L	University of Pennsylvania	USA	36
4	Schlosser, Rodney J	Medical University of South Carolina	USA	22
5	Frasnelli, Johannes	Université du Québec à Trois-Rivières	Canada	21
6	Pinto, Jayant M	University of Chicago	USA	20
7	Soler, Zachary M	Medical University of South Carolina	USA	19
8	Hansson, Bill S	Max Planck Institute for Chemical Ecology	Germany	18
9	Hopkins, Claire	King’s College London	England	18
10	Ben khemis, Ismahene	University of Monastir	Tunisia	15

### 3.4 Analysis of journals

**FIGURE 5:**
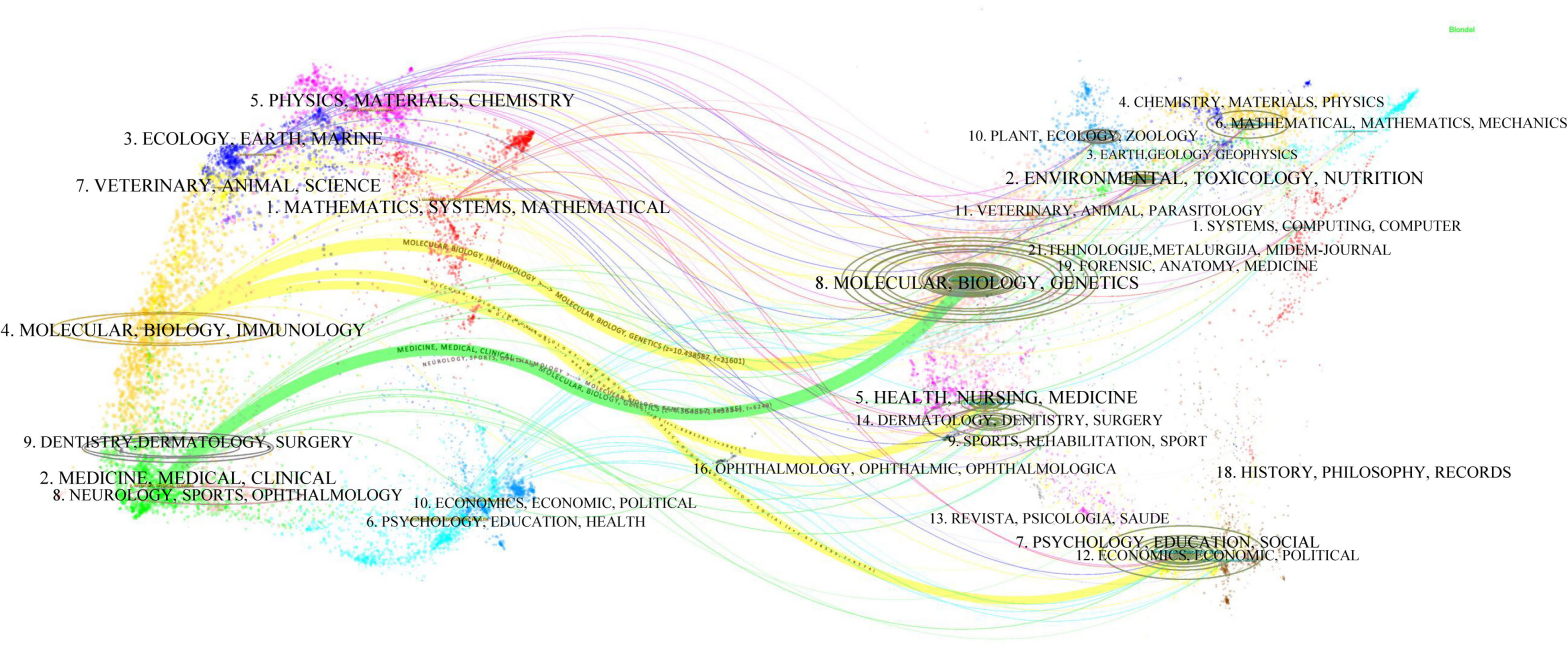
The dual-map overlay of journals.

Figure 5 is a double image overlay of journals, used to reflect the disciplinary flow and interaction between published journals and cited journals. The figure shows that research results published mainly in journals related to molecular biology, immunology, and clinical medicine are often cited by journals in fields such as molecular biology and genetics, health sciences, environmental sciences, psychology, and other social sciences.

Table 5 shows the top 10 journals with the highest publication volume in the field of OD mechanisms. *Scientific Reports* ranks first with 163 publications. The research findings are primarily published in multidisciplinary, life sciences, molecular sciences, neuroscience, and otorhinolaryngology journals from the USA, England, Germany, France, and Switzerland. These journals represent important publication platforms in this field, indicating that research on OD mechanisms spans both basic life sciences and clinical disciplines. Among the top 10 journals with the highest number of co-citations (Table 6), the list comprises 7 journals from the USA and 2 from England.

**TABLE 5:** The top 10 most productive journals.

Rank	Number of publication	Journal	Country/region	Impact factor (2025)
1	163	SCIENTIFIC REPORTS	England	3.9
2	109	PLOS ONE	USA	2.6
3	100	CHEMICAL SENSES	France	1.9
4	91	EUROPEAN ARCHIVES OF OTO-RHINO-LARYNGOLOGY	Germany	2.2
5	91	INTERNATIONAL JOURNAL OF MOLECULAR SCIENCES	USA	4.9
6	71	NATURE COMMUNICATIONS	England	15.7
7	63	LARYNGOSCOPE	USA	2
8	55	JOURNAL OF NEUROLOGY	Germany	4.6
9	53	ELIFE	England	N/A (no current JIF)
10	52	FRONTIERS IN NEUROLOGY	Switzerland	2.8

**TABLE 6:** The top 10 most productive co-cited journals.

Rank	Co-citation count	Journal	Country/region	Impact factor (2025)
1	3489	PLOS ONE	USA	2.6
2	2911	NATURE	England	48.5
3	2483	SCIENCE	USA	45.8
4	2198	CHEM SENSES	France	1.9
5	1976	CELL	USA	42.5
6	1968	NEURON	USA	15.3
7	1902	JOURNAL OF NEUROSCIENCE	USA	4
8	1871	PROCEEDINGS OF THE NATIONAL ACADEMY OF SCIENCES OF THE UNITED STATES OF AMERICA	USA	9.1
9	1684	NEUROLOGY	USA	9
10	1654	SCIENTIFIC REPORTS	England	3.9

### 3.5 Analysis of cited references and co-cited references

Table 7 presents the top 10 highly cited references in the field of OD mechanisms. These studies mainly address neurodegenerative diseases such as PD and the neurological effects of SARS-CoV-2 infection, including long COVID related manifestations. Among them, “Armstrong et al. (20), JAMA” ranks first with 2,386 citations. Most of the highly cited references published from 2020 onward appeared in high impact general medical, neuroscience, and life science journals. Table 8 displays the top 10 highly co-cited references in this field. Unlike the thematic diversity of highly cited papers, co-cited papers exhibit a high degree of focus, largely centered on COVID-19-related olfactory and nervous system mechanisms. “Mao et al. (21), JAMA Neurology” leads with 274 co-citations, highlighting the neurological manifestations and nervous system involvement in hospitalized patients with COVID-19. “Brann et al. (22), Science Advances” ranks second with 215 co-citations and provides a molecular-level explanation for clinically observed OD.

**TABLE 7:** Top 10 most cited references on the mechanisms of olfactory dysfunction.

Rank	Article title	Author	Published year	Journal	Count	Impact factor
1	Diagnosis and Treatment of Parkinson Disease: A Review	Armstrong, Melissa J.	2020	JAMA-JOURNAL OF THE AMERICAN MEDICAL ASSOCIATION	2386	55
2	Nervous system involvement after infection with COVID-19 and other coronaviruses	Wu, Yeshun	2020	BRAIN BEHAVIOR AND IMMUNITY	1525	7.6
3	Non-motor features of Parkinson disease	Schapira, Anthony H. V.	2017	NATURE REVIEWS NEUROSCIENCE	1410	26.7
4	Long covid-mechanisms, risk factors, and management	Crook, Harry	2021	BMJ-BRITISH MEDICAL JOURNAL	1341	43
5	SARS-CoV-2 is associated with changes in brain structure in UK Biobank	Douaud, Gwenaelle	2022	NATURE	1116	48.5
6	Real-time tracking of self-reported symptoms to predict potential COVID-19	Menni, Cristina	2020	NATURE MEDICINE	1039	50
7	Long COVID or post-COVID-19 syndrome: putative pathophysiology, risk factors, and treatments	Yong, Shin Jie	2021	INFECTIOUS DISEASES	920	2.3
8	Correlates of protection against symptomatic and asymptomatic SARS-CoV-2 infection	Feng, Shuo	2021	NATURE MEDICINE	920	50
9	Risk and predictors of dementia and parkinsonism in idiopathic REM sleep behavior disorder: A multicenter study	Postuma, Ronald B	2019	BRAIN	896	11.7
10	The emerging spectrum of COVID-19 neurology: Clinical, radiological and laboratory findings	Paterson, Ross W	2020	BRAIN	888	11.7

**TABLE 8:** Top 10 co-cited references on the mechanisms of olfactory dysfunction.

Rank	Article title	Author	Published year	Journal	Count	Impact factor
1	Neurologic Manifestations of Hospitalized Patients With Coronavirus Disease 2019 in Wuhan, China	Mao, Ling	2020	JAMA NEUROLOGY	274	21.4
2	Non-neuronal expression of SARS-CoV-2 entry genes in the olfactory system suggests mechanisms underlying COVID-19-associated anosmia	Brann, David H.	2020	SCIENCE ADVANCES	215	12.5
3	Olfactory and gustatory dysfunctions as a clinical presentation of mild-to-moderate forms of the coronavirus disease (COVID-19): a multicenter European study	Lechien, Jerome R.	2020	EUROPEAN ARCHIVES OF OTO-RHINO-LARYNGOLOGY	212	2.2
4	SARS-CoV-2 Cell Entry Depends on ACE2 and TMPRSS2 and Is Blocked by a Clinically Proven Protease Inhibitor	Hoffmann, Markus	2020	CELL	186	42.5
5	A first case of meningitis/encephalitis associated with SARS-Coronavirus-2	Moriguchi, Takeshi	2020	INTERNATIONAL JOURNAL OF INFECTIOUS DISEASES	170	4.3
6	Clinical features of patients infected with 2019 novel coronavirus in Wuhan, China	Huang, Chaolin	2020	LANCET	165	88.5
7	Evidence of the COVID-19 Virus Targeting the CNS: Tissue Distribution, Host-Virus Interaction, and Proposed Neurotropic Mechanisms	Baig, Abdul Mannan	2020	ACS CHEMICAL NEUROSCIENCE	157	3.9
8	Olfactory transmucosal SARS-CoV-2 invasion as a port of central nervous system entry in individuals with COVID-19	Meinhardt, Jenny	2021	NATURE NEUROSCIENCE	142	20
9	The neuroinvasive potential of SARS-CoV2 may play a role in the respiratory failure of COVID-19 patients	Li, Yan-Chao	2020	JOURNAL OF MEDICAL VIROLOGY	126	4.6
10	COVID-19-associated Acute Hemorrhagic Necrotizing Encephalopathy: Imaging Features	Poyiadji, Neo	2020	RADIOLOGY	124	15.2

### 3.6 Analysis of keywords

**FIGURE 6:**
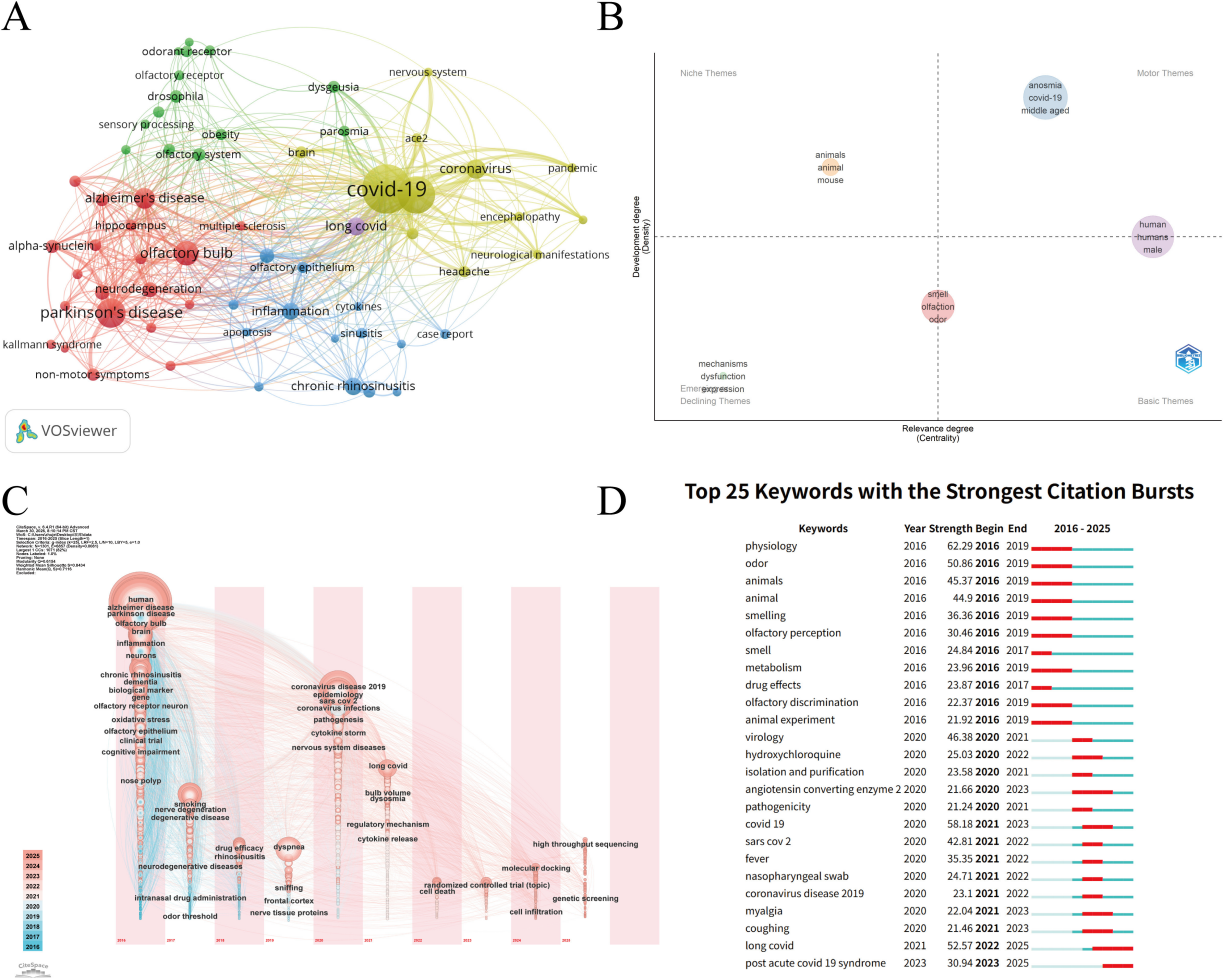
Keyword analysis. **(A)** Keyword clustering. **(B)** Thematic map. **(C)** Timezone view of keyword co-occurrence analysis. **(D)** Burst test of the keywords.

Keyword clustering analysis based on bibliometrics identified four distinct thematic groups in research on the mechanisms of OD. The red cluster centered on “Parkinson’s disease,” “Alzheimer’s disease,” and “olfactory bulb,” reflecting research on neurodegenerative diseases and central olfactory pathway involvement. The yellow cluster featured “COVID-19,” “ace2,” and “nervous system,” indicating OD associated with SARS-CoV-2 infection and its potential neurological involvement. The blue cluster comprised “chronic rhinosinusitis,” “inflammation,” and “olfactory epithelium,” representing inflammatory sinonasal disease and epithelial injury-related mechanisms. The green cluster encompassed terms such as “odorant receptor,” “olfactory system,” and “sensory processing,” highlighting olfactory perception, receptor-related biology, and basic signal transduction mechanisms (Figure 6A).

Figure 6B shows that COVID-19 related topics are in a relatively mature stage of development, and animal model research represents a relatively mature but specialized direction. The timeline shows that research hotspots in OD mechanisms have evolved from early neurodegenerative and inflammatory topics to COVID-19-related themes and, more recently, to long COVID, while regulatory mechanisms, cell death, molecular docking, and genetic screening have emerged as new frontiers (Figure 6C).

Keyword burst analysis (Figure 6D) further substantiates these phased transitions. In recent years, the intense and ongoing citation bursts for keywords such as “long COVID” and “post acute COVID 19 syndrome” strongly suggest that contemporary research is pivoting toward the mechanisms of persistent post-viral OD and its long-term clinical manifestations.

### 3.7 Analysis of clinical trial progress

In order to further explore the translation of OD mechanism research into clinical applications, we searched the PubMed database for clinical trials related to OD mechanisms to supplement the quantitative results and provide more practical references for researchers. We retrieved 34 results, and the exclusion of studies was independently verified by two authors to enhance reliability. After excluding all observational studies, non-English publications, and studies unrelated to the OD mechanism, 14 interventional clinical trials that met the criteria were included in the analysis (Figure 1 and Supplementary Table 1). Given the limited number of eligible trials, the following findings should be interpreted as an exploratory, descriptive synthesis rather than a formal systematic review.

Tracking the regulatory information of these 14 studies indicated that 5 studies provided formal clinical trial registration number (23–27). In contrast, the remaining 9 studies were ethically approved studies (28–36), although they were not registered on public clinical trial registration platforms. This is more common in early-stage, pilot, or non-drug-regulated intervention studies, and therefore, the lack of a registration ID was not used as an exclusion criterion. Due to the lack of traceable trial IDs or accessible protocol records in the other 9 studies, their methodological transparency is relatively limited, and compliance with the protocol cannot be independently verified.

Regarding interventional modalities, the included trials are categorized into pharmacological treatments, non-pharmacological therapies, surgical interventions, and mechanistic exploratory studies. Pharmacological interventions are the most prevalent; trials evaluating agents such as Vericiguat, glutamate diacetate (GLD), and palmitoylethanolamide and luteolin (PEA-LUT) reported notable, albeit varying, statistical improvements in TDI (Threshold, Discrimination, and Identification) scores (23, 33, 34). Non-pharmacological approaches, including olfactory training combined with transcranial direct current stimulation or acupuncture, also demonstrated promising therapeutic trends (28, 32). Conversely, surgical interventions have not yielded definitive overall benefits for OD (35, 36). Finally, select trials focused directly on mechanistic biomarkers, such as evaluating lactoferrin-related changes and DNA methylation in the nasal epithelium following RSV immunoprophylaxis (25, 26). Collectively, these diverse strategies reflect the active, multi-dimensional efforts in the field of OD.

## 4 Discussion

### 4.1 Summary of main findings

Over the past decade, research on the mechanisms of OD has exhibited an overall upward trend, profoundly accelerated by the COVID-19 pandemic. Although publication volumes have fluctuated slightly in the post-pandemic era, OD remains a high-priority research frontier.

The observed country-level pattern suggests that influence in OD mechanism research cannot be understood solely in terms of publication volume. Although the USA appears to be the principal driver of knowledge production and citation impact, several European countries seem to play a disproportionately important role in connecting the international research network. This difference implies that productivity and collaborative influence reflect distinct dimensions of scientific leadership. Overall, the global collaboration network appears to be centered on the USA, linking Europe and East Asia, with dense connections and close exchanges among the major countries.

At the institutional level, the prominent positions of the University of California System, CNRS, the Chinese Academy of Sciences, and several major European institutions indicate that this field is supported by both high productivity institutions and network bridging institutions. Although collaboration within major institutional clusters appears to be relatively close, the overall network structure suggests that strong cross-border collaboration remains an important driving force for progress in OD mechanism research.

Beyond geographical metrics, our bibliometric analysis highlights the foundational contributions of pioneering scholars. For instance, Thomas Hummel and his team not only led diagnostic consensus efforts but also pioneered the “Sniffin’ Sticks” olfactory training therapy, which systematically demonstrated for the first time that daily exposure to specific odors can improve olfactory function, making a significant Contribution to the field of OD (1, 37).

Although there is some overlap between the most productive journals and the most frequently co-cited journals, the two lists still reflect different tendencies. The most frequently co-cited journals are overall represented by highly influential journals such as *Nature*, *Science*, and *Cell*, indicating that the knowledge base of OD mechanism research is strongly rooted in broadly recognized biomedical and life science literature. In contrast, the most productive journals are represented by *Scientific Reports*, *PLOS One*, and *Chemical Senses*, suggesting that current studies are more actively disseminated through multidisciplinary and field-specific outlets. This pattern indicates that OD mechanism research has developed on the basis of influential foundational knowledge while continuing to expand through active publication in a broader range of journals. Therefore, in tracking this field, attention should be paid not only to seminal studies published in leading journals, but also to recent advances reported in core professional journals.

The most highly cited reference in the OD field provided a broad clinical overview of PD, emphasizing that hyposmia may precede motor symptoms and offering an important theoretical and clinical basis for recognizing OD as an early feature of PD (20). The top 10 highly co-cited references were published in journals spanning neurology, otorhinolaryngology, neuroscience, infectious diseases, virology, radiology, cell biology, and general medicine, highlighting the interdisciplinary knowledge base of OD mechanism research.

### 4.2 Etiology-specific neurobiological mechanisms

The keyword clustering analyses in the section “3 Results” reveal that research on OD mechanisms is primarily organized around several thematic clusters, including “odorant receptor,” “nervous system,” “COVID-19,” “inflammation,” “chronic rhinosinusitis,” and “Parkinson’s disease.” These thematic groupings not only reflect the main research hotspots over the past decade but also provide a bibliometric framework for discussing distinct etiological contexts of OD. Given this diversity, the following section systematically discusses the neurobiological mechanisms underlying OD according to different etiological categories, aiming to clarify how peripheral and central pathological processes converge across distinct disease backgrounds. It is important to note that while our bibliometric analysis highlights major research trends and key thematic clusters, it cannot by itself directly define specific disease mechanisms. Instead, the bibliometric results served as a macro-level navigational framework, guiding our subsequent qualitative synthesis. By manually reviewing the highly cited and pivotal papers within these clusters, we further interpreted the neurobiological features associated with different etiological contexts of OD. Therefore, the following discussion should be understood as a literature-based interpretive synthesis informed by bibliometric patterns, rather than as a direct algorithmic output of the bibliometric analysis.

#### 4.2.1 Etiologies related to peripheral olfactory epithelial damage

Regarding post-viral OD, current evidence suggests that different viruses damage the OE through distinct yet converging mechanisms. In COVID-19, SARS-CoV-2 appears not to directly infect OSNs. Instead, it predominantly acts on non-neuronal components, such as supporting cells within OE, indirectly precipitating OD by disrupting the local microenvironment and supportive functions (22). This pattern is highly consistent with the keyword clustering centered on COVID-19 and coronavirus. In contrast, some other viruses can directly infect and damage OSNs. Taking the influenza A virus as an example, animal studies have detected viral antigens in the OSNs of the olfactory mucosa, and apoptosis caused by OSNs infection may help limit virus transmission along the olfactory pathway (38). It is worth noting that in some olfactory virus models directly infected with OSNs, this peripheral damage is not limited to neuronal damage, but can also be further accompanied by nerve regeneration disorders, reduced olfactory sensory surface area, and metaplasia of olfactory mucosa to respiratory mucosa (39), which is related to the research hotspots of nervous system and pathogenesis. Overall, these findings indicate that post-viral peripheral OD may originate either from disturbances in non-neuronal epithelial components or from direct neuronal damage, both ultimately converging into impaired epithelial repair and persistent OD.

Moreover, the presence of terms such as chronic rhinosinusitis (CRS), inflammation, nasal polyps, and smoking in the keyword map indicates sustained research attention to chronic inflammatory diseases and environmental exposures in peripheral OD. The literature further suggests that peripheral OD is not solely attributable to post-viral damage but may also involve mechanisms associated with these conditions. Although CRS and environmental insults represent distinct pathogenic contexts, both can disrupt the homeostasis of the OE through continuous inflammation and epithelial barrier damage, thereby leading to OD (40, 41). Compared with CRS with nasal polyps (CRSwNP), CRS without nasal polyps (CRSsNP) exhibits greater inflammatory heterogeneity and less consistent type 2 predominance. Its pathogenesis often involves a complex interaction between epithelial barrier dysfunction and local immune responses (42). By comparison, OD in CRSwNP is more closely linked to type 2 inflammatory cytokines, such as IL-4, IL-5, and IL-13 (43). Research has shown that directly administering IL-4 into the nose of mice can lead to a rapid loss of their sense of smell. Under chronic type 2 inflammatory conditions, the mucociliary clearance and regenerative capabilities of the nasal epithelium may also be impaired (44). Meanwhile, environmental insults may aggravate this process through overlapping pathological pathways. Montgomery et al. proposed that the organic components of urban PM2.5 can induce strong transcriptomic reprogramming in human nasal mucociliary epithelium, activate inflammatory pathways, and promote mucus metaplasia-like remodeling (45). Cigarette smoke also elevates the levels of inflammatory cytokines such as TNF-α, compromising the epithelial barrier of the nasal mucosa. These effects are further exacerbated with prolonged exposure, thereby establishing a pathological foundation for the onset and progression of nasal diseases (46). Therefore, we deduce that CRS and environmental pollution may converge on shared pathological mechanisms, including epithelial barrier disruption, amplified inflammatory responses, and impaired tissue regeneration.

#### 4.2.2 Etiologies related to central olfactory pathway damage

Distinct from peripheral OD, central OD demonstrates a more profound association with structural and functional abnormalities along the intracerebral olfactory pathways. The prominence of key terms in the cluster analyses, such as OB, PD, AD, and alpha-synuclein, is consistent with the strong research focus on this aspect of OD. Central OD originates from systemic degenerative changes along the olfactory pathway at the neuroanatomical level, and its mechanism has specific manifestations in different neurodegenerative diseases (47). For example, in PD, this process is highly correlated with the dissemination pathway of alpha-synuclein: pathological changes begin in primary centers such as the OB/preorhinal nucleus and follow the pathway described by the Braak hypothesis to spread upward to higher brain regions such as the amygdala and temporal lobe (48). Similarly, in the very early stages of AD, although significant structural atrophy has not yet occurred, it has been confirmed that the functional network connecting the primary olfactory cortex and hippocampus is weakened, which constitutes the fundamental reason for the early decline in the association between olfactory and memory functions (49). Collectively, these findings identify central OD as a critical mechanistic clue for detecting early neurodegenerative changes. In addition to intrinsic neurodegenerative processes, environmental exposures such as PM2.5 may further increase central olfactory vulnerability (50). PM2.5 may enter the central nervous system through two main pathways: the blood-brain barrier and olfactory neurons. It can increase the risk of AD and PD by mechanisms such as oxidative stress, neuroinflammation, abnormal activation of microglia, and lipid metabolism disorders (51). This suggests that central OD reflects not only intrinsic neurodegeneration but also more extensive external invasion.

Post-traumatic olfactory dysfunction (PTOD) is a common consequence of traumatic brain injury (TBI), and is related to keywords such as brain and nerve degeneration. Although trauma can affect multiple levels of the olfactory system, persistent PTOD and related symptoms may be more associated with persistent abnormalities in central olfactory related brain regions (52). Mechanistically, this central component can manifest as bleeding in the olfactory cortex region and may be accompanied by secondary neurodegenerative changes, thereby affecting multiple levels of the olfactory system (53, 54). The study showed that TBI patients with olfactory loss had reduced OB volumes, and patients with functional anosmia additionally exhibited lower gray matter density in brain areas related to olfaction (55). Not only that, but these secondary changes may be related to sensory deprivation. The continuous loss of olfactory input provides an important basis for the sensory deprivation hypothesis (56). As Galliano et al. demonstrated in animal models, even a brief (1-day) sensory deprivation without damaging the OE is sufficient to trigger significant structural and functional plasticity of dopaminergic neurons in the OB (57). This observation echoes the hotspot terms neurodegeneration, neurons, and OB, and implies that, in PTOD patients with clear physical injury, the cascade of degenerative changes triggered by the loss of sensory input may be more rapid and profound.

#### 4.2.3 Etiologies related to developmental, metabolic, and multisystem factors

As indicated by the gene, OB and kallmann syndrome in the keyword map, compared with PTOD, congenital olfactory dysfunction (COD) mainly reflects developmental and genetic abnormalities of the olfactory system (58, 59). Structurally, patients with COD typically present with an absent or hypoplastic OB and a shallow olfactory sulcus. Furthermore, they also show increased gray and white matter volumes in brain areas related to sensory integration and memory (59). This suggests that COD may not only be due to underdeveloped olfactory structures, but also accompanied by abnormalities in related functional networks in the brain. In addition to anatomical evidence, genetic factors may also be involved in the occurrence of COD, as mutations in genes such as CNGA2 and TENM1 have been linked to it (60, 61). Moreover, this mechanistic complexity is particularly evident in syndromic COD, which can arise from diverse syndrome specific processes, including developmental defects, sensory signaling abnormalities, and dysfunction of olfactory cilia (62). Taken together, COD is more likely to reflect a broad abnormality in the formation and organization of the olfactory system.

In addition to these development-related mechanisms, the keywords further highlight terms such as inflammation, oxidative stress, and obesity, which may suggest multiple systemic mechanisms related to metabolic disorders. In this context, selecting representative metabolic diseases can further illustrate how systemic homeostasis imbalance is involved in olfactory damage. Chronic kidney disease is a typical example, in which OD is common and may be caused by multiple mechanisms, including OE damage from chronic inflammation, impaired neuronal and cognitive processing, hyperprolactinemia related dysregulation, and toxin related neurodegeneration (63). In particular, uremic toxins can induce damage to the OE, OB, and central olfactory pathway (64), further highlighting their extensive toxic effects on multiple levels of the olfactory system.

Poor glycemic control may also be a risk factor associated with OD (65). Studies have shown that persistent hyperglycemia in adult diabetic rats is associated with reduced diameters of OB glomeruli and mitral cells (66). Meanwhile, at the central level in humans, patients with type 2 diabetes mellitus not only exhibit reduced cerebral blood flow perfusion in multiple olfactory related brain regions, but also show significant abnormalities in cerebral blood flow connectivity (67). Furthermore, the degeneration of rhinencephalon structures in patients with type 1 diabetes is also closely related to diabetic peripheral neuropathy (68), suggesting that OD may manifest as part of a widespread metabolic neurodegenerative process.

Obesity is closely related to metabolic disorders of diabetes and is also another important influencing factor of OD (69, 70). Evidence suggests that, in terms of structural morphology, the OB volume of obese individuals is significantly smaller than that of normal weight individuals (71). At the metabolic level, obesity related OD is associated with an imbalance in hormone homeostasis, characterized by increased leptin, insulin, glucose, and HOMA-IR (an index of insulin resistance), as well as a decrease in ghrelin and IGF-1 (insulin-like growth factor 1) levels (72). This reflects the long-term interference of sustained metabolic disorders on the homeostasis of the olfactory system.

Overall, OD related to metabolic diseases may result from multilevel damage extending from the peripheral olfactory pathway to the central olfactory pathway, caused by the combined effects of chronic inflammation, toxin accumulation, hormonal and insulin signaling dysregulation, and central nervous system degeneration.

### 4.3 Core molecular signaling pathways

**FIGURE 7:**
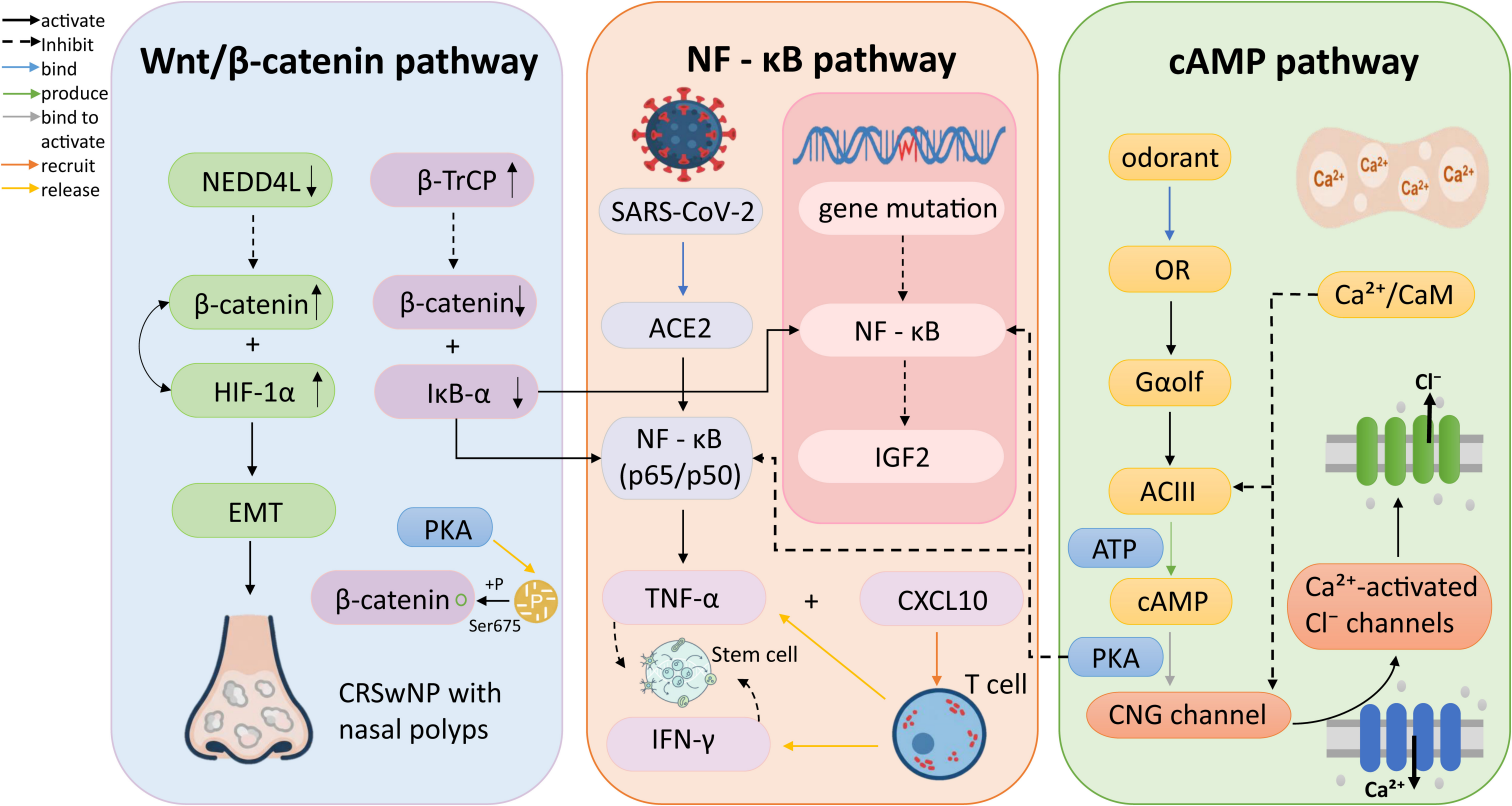
Schematic illustration of the crosstalk between Wnt/β-catenin, NF-κB, and cAMP pathways in olfactory dysfunction. (Left) Downregulation of NEDD4L leads to accumulation of β-catenin and HIF-1α, promoting epithelial–mesenchymal transition (EMT) and nasal polyp formation. (Middle) SARS-CoV-2 infection or genetic mutations activate the NF-κB pathway, inducing pro-inflammatory mediators (TNF-α, CXCL10, IFN-γ) in the olfactory epithelium and potentially impairing stem cell–mediated neuronal regeneration. (Right) In olfactory transduction, odorant binding to ORs activates the Gαolf–ACIII–cAMP cascade, triggering Ca^2+^ influx and Ca^2+^-activated Cl efflux to amplify sensory signals. Notably, protein kinase A (PKA) acts as a central crosstalk mediator by suppressing NF-κB-driven inflammation while phosphorylating β-catenin at Ser675, thereby influencing tissue remodeling and olfactory function. ACE2, angiotensin-converting enzyme 2; ACIII, adenylyl cyclase 3; ATP, adenosine triphosphate; β-TrCP, beta-transducin repeat-containing protein; cAMP, cyclic adenosine monophosphate; Ca^2 +^, calcium ion; CaM, calmodulin; Cl, chloride ion; CNG channel, cyclic nucleotide-gated channel; CRSwNP, chronic rhinosinusitis with nasal polyps; CXCL10, C-X-C motif chemokine ligand 10; EMT, epithelial–mesenchymal transition; Gαolf, olfactory-specific G protein alpha subunit; HIF-1α, hypoxia-inducible factor 1 alpha; IFN-γ, interferon gamma; IGF2, insulin-like growth factor 2; IκB-α, nuclear factor of kappa light polypeptide gene enhancer in B-cells inhibitor, alpha; NEDD4L, neural precursor cell expressed developmentally downregulated protein 4-like; NF-κB, nuclear factor kappa B; OR, olfactory receptor; PKA, protein kinase A; SARS-CoV-2, severe acute respiratory syndrome coronavirus 2; Ser675, serine residue at position 675; TNF-α, tumor necrosis factor alpha.

Although the etiological background of OD is diverse, the keyword clustering results still suggest several recurring process-level themes across different disease contexts, particularly inflammatory responses, receptor-related olfactory signaling, epithelial injury and repair, and neurodegenerative changes involving the central olfactory pathway. Based on these bibliometric patterns and further literature synthesis, we selected three representative signaling pathways for interpretive discussion: the NF-κB pathway that drives inflammatory responses, the cAMP pathway that mediates classical olfactory conduction, and the Wnt/β-catenin pathway that regulates stem cell regeneration and repair (73–75) (Figure 7). These pathways were used as literature-based mechanistic examples to interpret the major themes identified in the keyword network, rather than as direct outputs of bibliometric clustering.

The NF-κB pathway exhibits dual roles in the peripheral OE and olfactory center, respectively. For example, after SARS-CoV-2 virus invades supporting cells, it activates NF-κB (p65/p50). NF-κB entering the nucleus initiates transcription of genes such as TNF-α (injury signal) and CXCL10 (recruitment signal). CXCL10 recruits T cells, and the IFN-γ and TNF-α released by T cells act together on stem cells, preventing them from regenerating into new neurons, resulting in the inability to restore olfactory function (76, 77). In neurodegenerative diseases such as AD, genetic mutations in genes such as SHARPIN disrupt the activation of NF-κB in neurons, leading to the inability to produce the critical “protective protein” IGF2. As a result, neurons in the olfactory center (such as the entorhinal cortex) may become vulnerable and undergo progressive degeneration, potentially contributing to brain atrophy and functional loss (78, 79).

Moreover, the cAMP pathway serves as a crucial execution pathway for olfaction. Boccaccio et al. proposed a complete molecular chain from odor molecules to electrical signals: odor molecules bind to ORs, which activate the olfactory-specific G protein Gαolf. Gαolf then activates ACIII, converting ATP into cAMP. cAMP directly binds and opens cyclic nucleotide-gated channels (CNG channels). The opening of CNG channels leads to Ca^2 +^ influx, which subsequently activates calcium-activated chloride channels (Ca^2 +^-activated Cl^–^ channels), amplifying the electrical signal through Cl^–^ efflux. The influx of Ca^2 +^ binds to calmodulin (CaM), and the resulting complex inhibits ACIII and CNG channels, thereby achieving olfactory adaptation (80). Meanwhile, ANO2 (Anoctamin 2, also known as TMEM16B) is a calcium activated chloride ion channel in the cilia of OSNs, which may play a key role in amplifying olfactory signals (81). It is noteworthy that in the olfactory signaling pathway, the key molecule calcium Ca^2 +^ can both enhance signals by activating Cl^–^ channels and suppress the pathway through negative feedback, enabling neurons to adapt to persistent odor stimuli (75). This suggests that beyond pathway disruption, OD may arise from internal homeostatic imbalances, leading to nuanced clinical deficits such as altered sensitivity or perception.

Meanwhile, the maintenance and repair of olfactory function heavily rely on the regenerative capacity of adult stem cells (ASCs) within the OE. It is reported that the Wnt signaling pathway plays a crucial role in maintaining ASC stemness, which is beneficial for the generation of mouse ASC-derived cells. Meanwhile, LGR5 (Leucine-rich G repeat-containing protein-coupled receptor 5), as a key member of the Wnt signaling pathway, serves as a crucial marker for adult stem cells in the OE. Experimental results have shown that it is these LGR5^+^ stem cells that ensure the long-term expansion of mouse olfactory epithelial organoids in Rspondin1 based cultures (82). It is noteworthy that activating the Wnt/β-catenin pathway also has positive implications for the treatment of PD and AD (73). However, when the Wnt/β-catenin pathway is abnormally activated, such as in CRSwNP with nasal polyps, the expression of E3 ubiquitin ligase NEDD4L is downregulated, leading to the accumulation of its substrates β-catenin and HIF-1α that cannot be effectively degraded. A positive feedback loop is formed between β-catenin and HIF-1α, which together may promote epithelial-mesenchymal transition (EMT), subsequently contributing to the formation of nasal polyps (83, 84).

In addition, E3 ubiquitin ligase β-TrCP can simultaneously degrade β-catenin and IκB-α. When Wnt signaling activates its own pathway, it can enhance the degradation of IκB-α by upregulating β-TrCP, thereby enhancing the transcriptional activation of NF-κB (85). Meanwhile, long-term excessive Wnt signaling can activate and drive EMT (86). Both jointly disrupt the olfactory epithelial microenvironment, causing obstruction of olfactory conduction and instability of the neuroepithelium.

Furthermore, cAMP is an important regulatory factor for macrophage function and phenotype, and Lipoxin A4 in its pathway has a repairing effect on the airway epithelial barrier. And the regulatory effect of cAMP on pro-inflammatory mediators was first mediated by PKA, which can inhibit NF-κB-induced gene transcription (87). Jin et al. proposed that the cAMP-PKA-NF-κB pathway regulates the TNF-α-induced MUC-1 protein levels during RSV infection in A549 cells. Therefore, upregulating cAMP/PKA can inhibit the upregulation of NF-κB-driven mucin (88), which may improve mucociliary clearance function, thereby aiding in olfactory transmission.

At the same time, PKA can directly phosphorylate β-catenin, and this phosphorylation is an activation signal that enhances its transcriptional activity. Cipher mediated β-catenin Ser675 phosphorylation may be a cascade between Wnt signaling and cAMP/PKA pathway (89, 90). This means that in specific cases of OD, if one wants to increase cAMP/PKA for anti-inflammatory purposes, it may activate the Wnt/β-catenin pathway, which may exacerbate tissue remodeling associated with olfactory loss.

In summary, these three major signaling pathways together construct a complex regulatory network that drives the pathophysiological processes of OD from the peripheral to the central level.

### 4.4 Clinical implications

Based on this exploratory synthesis of a limited number of clinical trials, non-pharmacological therapies such as transcranial direct current stimulation combined with olfactory training and acupuncture have shown some potential (28, 32), suggesting that the recovery of olfactory function may benefit from sensory retraining and neural plasticity regulation. In contrast, the lack of significant benefits from surgical interventions implies that their clinical application should still be cautious at this stage (35, 36). In addition, mechanistic studies such as those on lactoferrin supplementation and RSV immunoprophylaxis have indicated that future clinical trials should place greater emphasis on the integration of mechanism research and efficacy evaluation (25, 26).

As for pharmacological treatments, PEA-LUT, Gabapentin, surfactant solution, Pentoxifylline, Vericiguat, Omalizumab, and GLD were included in the study, and their implementation methods mainly include oral administration, nasal irrigation, and subcutaneous injection (23, 24, 27, 29, 31, 33, 34). Among them, GLD adopts a relatively special administration route of local intranasal administration, and improvements in TDI scores were observed (34). This result provides preliminary clinical evidence for the application of intranasal administration in OD intervention. Beyond topical GLD, other relatively conventional intranasal approaches, such as intranasal insulin, have also shown preliminary functional benefits (91). Moreover, another study employed a micropipette to locally instill CGFs (concentrated growth factors) into both nasal cavities and maintained the supine position after administration. The results indicated that this approach could promote the repair and regeneration of the OE (92). In contrast to these relatively conventional local approaches, intranasal administration of stem cell-derived exosomes can achieve nose-to-brain transport through the olfactory and trigeminal nerve pathways, thereby exerting regulatory effects on the central nervous system (93). However, intranasal administration remains largely at an early stage of research and translation stage, and more advanced targeted delivery systems will be essential for its further clinical translation.

Overall, the field of OD mechanisms is still in the early stage of evidence accumulation, and high-quality confirmatory studies are still lacking. There is still considerable room for further exploration in the future.

### 4.5 Limitations

This research has several limitations. Firstly, the literature data in this study were mainly derived from WoSCC and Scopus for bibliometric analysis, while PubMed was only used to supplement the clinical intervention evidence. Although this dual-database integration strategy covered a broad scope, other databases such as Embase were not included for technical reasons, which may have led to the omission of some relevant foundational or regional literature, thereby introducing potential database selection bias. Secondly, the number of eligible interventional clinical studies included in this study was limited (only 14), not due to constraints in the retrieval strategy or screening method, but because there remains a scarcity of clinical trials related to OD mechanisms. Moreover, this part of the study was designed as an exploratory and descriptive synthesis rather than a formal systematic review, which makes it difficult to comprehensively reflect the overall clinical translation status of OD mechanism research. Finally, the discussion of OD-related mechanisms was not fully comprehensive. Although the bibliometric results provided a macro level framework, the mechanistic interpretation still relied in part on a qualitative manual review of representative studies, which may have introduced a degree of interpretive subjectivity. Future studies should therefore incorporate broader data sources, more rigorous evidence synthesis strategies, and larger, better designed experimental studies to further elucidate the exact mechanisms underlying OD.

## 5 Conclusion

This study combined bibliometric analysis and clinical evidence synthesis to explore the mechanism of OD. The results suggest that olfactory epithelial injury and repair, inflammatory mechanisms, receptor related olfactory signaling, and degeneration of the central olfactory pathway are major research hotspots in the study of OD mechanisms across different etiological backgrounds. At the molecular level, NF-κB, cAMP, and Wnt/β-catenin appear to represent key signaling pathways involved in the interactions among inflammation, signal transduction, and neural regeneration. Therefore, future research may benefit from prioritizing mechanism-based intervention measures and multimodal treatment strategies targeting these pathways in OD. However, given the complexity of these mechanisms, it is necessary to continue exploration to overcome this key challenge of developing effective clinical intervention measures.

## Data Availability

The original contributions presented in this study are included in the article/Supplementary material, further inquiries can be directed to the corresponding authors.
