# Supplementary material for: Neurobiological mechanisms of olfactory dysfunction: a ten-year bibliometric and visualization analysis
Source: Front Med (Lausanne). 2026 May 13;13:1812327. doi: 10.3389/fmed.2026.1812327 (PMC13212058; doi:10.3389/fmed.2026.1812327)
Supplement: Supplementary file 1 [file Table_1.docx]

**Table S1 Summary of Interventional Clinical Trials in the Field of Olfactory Dysfunction Mechanisms***

| **First-Author** | **Year** | **Participants** | **Treatment** | **Effects** | **Study limitations** | **Trial registration** | **Reference** |
| --- | --- | --- | --- | --- | --- | --- | --- |
| Pietro De Luca | 2022 | 69 patients with Long COVID and chronic olfactory dysfunction (43 female, 26 male; mean age 40.6) | PEA-LUT | PEA-LUT improved odor identification scores and reduced parosmia severity. Patients receiving the combination with olfactory training reported improvements in mental clouding as well. | Groups differed in size; no pure placebo arm; several outcomes relied on subjective reporting; modest overall sample size | NCT04853836 (ClinicalTrials.gov) | (1) |
| [Lucilla Vestito](https://pubmed.ncbi.nlm.nih.gov/?term=Vestito+L&cauthor_id=40348180) | 2025 | 52 patients with persistent COVID-19 hypo/anosmia completed the RCT; 35 in the experimental group and 17 in the placebo group; mean age 46.3 years; 62% female | Anodal tDCS over the left prefrontal cortex plus olfactory training, Sham tDCS plus olfactory training | The experimental group showed significant and sustained improvement in both subjective and objective olfactory measures versus placebo. Mean VAS-smell increased from 2.56 to 4.70 and mean SST from 6.97 to 9.69 at treatment end (both P<0.001). Overall, 77% significantly improved and 63% achieved normosmia; benefits persisted at 1- and 6-month follow-up. | The odor identification subtest rather than the full Sniffin' Sticks battery was used; not all possible predictors of persistence/recovery were assessed (e.g., comorbidities, nasal congestion, parosmia/phantosmia, viral load); small sample size with group imbalance in smell-related jobs; no functional neuroimaging was performed to identify responders. | Not registered (Ethics approval: Liguria Territorial Ethics Committee 244/2023 - DB id 13157) | (2) |
| [Ashna Mahadev](https://pubmed.ncbi.nlm.nih.gov/?term=Mahadev+A&cauthor_id=37733356) | 2023 | 68 adults with post-COVID-19 olfactory dysfunction of at least 3 months’ duration were randomized 1:1 to gabapentin or placebo (34 per arm); mean age 43 years; 75% female | Gabapentin, Placebo | Gabapentin showed no statistically significant or clinically meaningful benefit over placebo. After the 8-week fixed-dose phase, responder rates were 44.4% in the gabapentin group and 46.2% in the placebo group; no significant differences were observed in UPSIT, ODOR, NASAL-7, or other secondary outcomes. No serious adverse events occurred. | Small pilot sample; substantial dropout and unbalanced completion between groups, especially before treatment initiation in the gabapentin arm; predominantly White and female single-center cohort limiting generalizability; virtual screening without in-person examination may have missed other causes of olfactory dysfunction; exact duration of olfactory dysfunction was not measured; longer treatment effects were not assessed. | NCT05184192  (ClinicalTrials.gov) | (3) |
| [Justin H Turner](https://pubmed.ncbi.nlm.nih.gov/?term=Turner+JH&cauthor_id=28558139) | 2017 | 33 healthy volunteers (7 male, 26 female; mean age 37.6 years) with normosmic baseline results were randomized in a controlled crossover study | Surfactant nasal irrigation, Buffered isotonic saline irrigation | Surfactant irrigation was associated with a small but statistically significant reduction in mean SIT-40 score (−1.5 points; P=0.012) versus no change with saline. A clinically meaningful smell reduction (≥4 points) occurred in 18% after surfactant versus 3% after saline (P=0.046). Moderate or severe congestion was also more frequent with surfactant (29% vs 6%; P=0.021). Olfactory decline appeared reversible in follow-up cases. | Small single-center sample; non-blinded design; conducted in healthy volunteers rather than CRS patients, limiting generalizability; incomplete diary return in some subjects; female predominance in the sample; compensation may have reduced apparent treatment intolerance. | Not registered (IRB-approved at Vanderbilt University Medical Center) | (4) |
| [Agnieszka Sabiniewicz](https://pubmed.ncbi.nlm.nih.gov/?term=Sabiniewicz+A&cauthor_id=40317228) | 2025 | 128 normosmic adults aged 50–85 years completed the study; 19 assigned to pleasant-odor OT, 25 to unpleasant-odor OT, 28 to physical exercise, and 35 to control; 105 women and 23 men | Pleasant-odor olfactory training, Unpleasant-odor olfactory training, Physical exercise, Control | Neither olfactory training nor physical exercise significantly improved olfactory function. However, both OT groups showed significant improvement in verbal fluency, with the strongest effect in the unpleasant-odor OT group; physical exercise also improved verbal fluency, but more weakly. A significant reduction in depression severity was observed only in the pleasant-odor OT group with higher baseline depressive symptoms. | Baseline differences in verbal fluency and depression across groups may have influenced results; all dropouts occurred in the unpleasant-odor OT group and compliance was lower in that group; training duration differed between groups; physical activity was not tightly monitored; some data violated assumptions of normality/homogeneity, so repeated-measures ANOVA should be interpreted cautiously. | Not registered (c Wroclaw, EK 2022/ERBOA) | (5) |
| Katherine L. Whitcroft | 2020 | 6 patients with post-traumatic olfactory dysfunction (4 male, 2 female; mean age 43 years) | Oral Pentoxifylline | No meaningful improvement in olfactory function was detected. TDI and individual sub-scores showed only minimal fluctuations, and none reached statistical or clinical significance. Treatment was well tolerated. | Very small sample (pilot study), absence of randomization or control group, and short treatment duration | Not registered; ethics approval mentioned (no ID) | (6) |
| [Tanja Drews](https://pubmed.ncbi.nlm.nih.gov/?term=Drews+T&cauthor_id=34032906) | 2022 | 60 patients with postinfectious smell loss were randomized; 30 assigned to verum acupuncture and 30 to sham acupuncture; 23 male and 37 female; mean age 64.7 years | Verum acupuncture, Sham acupuncture | Compared with sham acupuncture, verum acupuncture was associated with significantly greater improvement in overall olfactory function as measured by the TDI score (P=0.039), mainly driven by improvement in odor discrimination (P=0.003). Clinically relevant TDI improvement (>5.5 points) occurred in 20% of the verum group versus 10% of the sham group, although this difference was not statistically significant. Better response was associated with shorter disease duration. | Single-center study; relatively small sample size; patient-blinded but not clearly fully double-blinded; no long-term follow-up to determine durability of benefit; clinically significant improvement was observed only in a minority of patients; underlying mechanism remains uncertain. | Not registered (Ethics approval: EK 78032012) | (7) |
| [Aili Wang](https://pubmed.ncbi.nlm.nih.gov/?term=Wang+A&cauthor_id=30182113) | 2018 | 19 cancer patients with chemotherapy-induced taste and smell abnormalities were enrolled; 12 patients with stable/reproducible salivary proteomes were included in proteome and mineral analyses; 12 healthy subjects were also enrolled as controls for biomarker analyses | Oral lactoferrin supplementation (250 mg/tablet, one tablet three times daily for 30 days) | Taste and smell abnormality scores decreased after lactoferrin supplementation, with significant reductions in taste, smell, and total abnormality scores by 30 days post-supplementation. Lactoferrin significantly reduced salivary iron concentration and increased several salivary immune-related proteins, supporting feasibility as a treatment for chemotherapy-induced taste and smell abnormalities. | Small sample size; only 12 cancer patients were included in detailed proteomic/mineral analyses; no randomized placebo-controlled design; heterogeneous cancer types and chemotherapy regimens; outcomes relied partly on self-reported symptom scores; mechanism remains preliminary and requires confirmation in larger controlled trials. | NCT01596634  (ClinicalTrials.gov) | (8) |
| Adnan Alharbi | 2024 | 88 patients with persistent post-infectious anosmia (44 female, 44 male; mean age ~44 years). | Vericiguat、 Placebo | Vericiguat produced statistically significant gains in threshold, discrimination, and identification scores compared with placebo. However, the mean TDI improvement (+3.19) did not reach the predefined threshold for clinical relevance (+5.5). | Pilot-level sample size and notable dropout rate (particularly in the placebo arm). Observed improvements, although statistically significant, were modest. Mechanistic basis remains uncertain. | Not registered (IRB:DFM-IRB00012367-23-07-008) | (9) |
| [Cheng-Jian Xu](https://pubmed.ncbi.nlm.nih.gov/?term=Xu+CJ&cauthor_id=34473906) | 2021 | 429 preterm infants were originally randomized in the MAKI trial to palivizumab (n=214) or placebo (n=215); nasal methylation analysis at age 6 included 274 children after quality control (132 palivizumab, 142 placebo) | Palivizumab RSV immunoprophylaxis, Placebo | Infant RSV immunoprophylaxis had significant long-term effects on global nasal epithelial DNA methylation at age 6. The intervention-associated methylation pattern was enriched for pathways related to sensory perception of smell, epidermal growth factor receptor signaling, and T-cell differentiation. Three CpG sites (annotated to GLB1L2, SC5D, and BPIFB1) were differentially methylated at genome-wide significance, but these epigenetic changes were not associated with asthma at age 6. | Secondary mechanistic follow-up analysis rather than a primary trial efficacy report; no independent replication cohort; cell-type composition in nasal brushes could not be directly defined and strongly influenced global methylation results; study was single-blinded; methylation changes were not directly linked to gene expression; global methylation analyses were exploratory. | ISRCTN73641710 | (10) |
| [Cecelia Damask](https://pubmed.ncbi.nlm.nih.gov/?term=Damask+C&cauthor_id=34382434) | 2022 | 265 patients with CRSwNP from the pooled POLYP 1 and POLYP 2 phase III trials were included (omalizumab n=134; placebo n=131); mean age about 50 years; approximately 64% male | Omalizumab, Placebo | Omalizumab consistently improved clinical and patient-reported outcomes versus placebo across prespecified subgroups defined by blood eosinophil count, prior sinonasal surgery, asthma, and aspirin sensitivity. Improvements were observed in NCS, NPS, SNOT-22, TNSS, and UPSIT at week 24, suggesting broad efficacy regardless of these baseline characteristics. | Majority of participants were White and male, limiting generalizability; some subgroup sizes remained small despite pooling; no standardized NPS grading system across centers; prior surgery subtype and its effect on smell recovery could not be differentiated; this was a pooled subgroup/secondary analysis rather than a primary trial report. | NCT03280550; NCT03280537  (ClinicalTrials.gov) | (11) |
| Majed A. Algarni | 2025 | 66 adults with post-COVID-19 olfactory dysfunction persisting >6 months (GLD: n=33; saline: n=33). | GLD | Significant improvements were observed in threshold, discrimination, and identification scores (all P < .0001), accompanied by reduced nasal calcium levels. Despite these findings, the overall TDI change (+2.84) remained below the clinical significance criterion (MCID 5.5) | Sample size limited; nasal secretions were not collected directly from the olfactory cleft; sampling time points varied;parosmia/phantosmia not evaluated; spontaneous recovery cannot be excluded. Study power slightly below target (0.75). | Not registered (IRB:DFM-IRB00012367-24-12-008) | (12) |
| [Courtney T Chou](https://pubmed.ncbi.nlm.nih.gov/?term=Chou+CT&cauthor_id=32921136) | 2020 | 31 patients undergoing endoscopic endonasal approach for sellar pathology were enrolled; 16 underwent EEA without nasoseptal flap and 15 with nasoseptal flap; among the NSF group, 8 were randomized to dominant-side harvest and 6 to non-dominant-side harvest; mean age 47.2 years | Nasoseptal flap elevation during EEA, No nasoseptal flap control; dominant-side NSF harvest, non-dominant-side NSF harvest | NSF use did not significantly affect binarial olfaction, uninarial olfaction, or rhinologic quality of life compared with controls. NSF elevation resulted in a small non-significant decrease in UPSIT scores (4% overall), and dominant-side harvest also did not significantly worsen outcomes versus non-dominant-side harvest. | Sample size was small and underpowered to detect small effects; NSF use itself was not randomized, only harvest side was randomized; middle turbinate resection was not controlled; possible recall bias on repeated olfactory testing; criteria for defining dominant versus non-dominant uninarial olfaction were novel and not yet validated. | Not registered (University of Pittsburgh IRB approval reported) | (13) |
| [Cassandra L Puccinelli](https://pubmed.ncbi.nlm.nih.gov/?term=Puccinelli+CL&cauthor_id=30657649) | 2019 | 22 adults undergoing transnasal endoscopic skull-base surgery with septal incisions were analyzed; 10 randomized to cold knife and 12 to monopolar cautery; mean age 50.2 years; 54% female | Cold knife upper septal limb incision, Monopolar cautery upper septal limb incision | No significant short-term or long-term differences in olfactory outcomes were found between groups. Preoperative, 3-month, and 12-month postoperative UPSIT scores were similar, and there was no significant difference in SNOT-22 smell/taste scores at any time point. Subjective smell reduction showed a nonsignificant transient decline at 3 months that appeared to recover by 1 year. | Small single-center sample; 24% of initially enrolled patients were excluded because of incomplete follow-up, introducing possible selection bias; heterogeneous surgical techniques and variable use of instrumentation for septal incisions other than the upper limb incision may have confounded results; surgical variability across flap types and adjunct procedures limits generalizability. | Not registered (IRB 15-008207) | (14) |

*The clinical trials are obtained from PubMed, and the search process for these data does not fully comply with the requirements of a systematic review.

**References**

1. De Luca P, Camaioni A, Marra P, Salzano G, Carriere G, Ricciardi L, et al. Effect of Ultra-Micronized Palmitoylethanolamide and Luteolin on Olfaction and Memory in Patients with Long COVID: Results of a Longitudinal Study. *Cells* (2022) 11:2552. doi: 10.3390/cells11162552

2. Vestito L, Ponzano M, Mori L, Trompetto C, Bandini F, Canta R, et al. A randomized controlled trial of anodal transcranial direct current stimulation (A-tDCS) and olfactory training in persistent COVID-19 anosmia. *Brain Stimulat* (2025) 18:1106–1112. doi: 10.1016/j.brs.2025.04.023

3. Mahadev A, Hentati F, Miller B, Bao J, Perrin A, Kallogjeri D, et al. Efficacy of Gabapentin For Post–COVID-19 Olfactory Dysfunction: The GRACE Randomized Clinical Trial. *JAMA Otolaryngol Neck Surg* (2023) 149:1111. doi: 10.1001/jamaoto.2023.2958

4. Turner JH, Wu J, Dorminy CA, Chandra RK. Safety and tolerability of surfactant nasal irrigation. *Int Forum Allergy Rhinol* (2017) 7:809–812. doi: 10.1002/alr.21959

5. Sabiniewicz A, Reichert A, Oleszkiewicz A, Hähner A, Hummel T. Effects of physical exercise and olfactory training with pleasant and unpleasant odors on verbal fluency and depression. *Chem Senses* (2025) 50:bjaf013. doi: 10.1093/chemse/bjaf013

6. Whitcroft KL, Gudziol V, Hummel T. Short-Course Pentoxifylline Is Not Effective in Post-Traumatic Smell Loss: A Pilot Study. *Ear Nose Throat J* (2020) 99:58–61. doi: 10.1177/0145561319840888

7. Drews T, Hummel T, Rochlitzer B, Hauswald B, Hähner A. Acupuncture is associated with a positive effect on odour discrimination in patients with postinfectious smell loss—a controlled prospective study. *Eur Arch Otorhinolaryngol* (2022) 279:1329–1334. doi: 10.1007/s00405-021-06872-9

8. Wang A, Duncan SE, Lesser GJ, Ray WK, Dietrich AM. Effect of lactoferrin on taste and smell abnormalities induced by chemotherapy: a proteome analysis. *Food Funct* (2018) 9:4948–4958. doi: 10.1039/C8FO00813B

9. Alharbi A, Abdelazim MH, Alshammari AS, Algarni MA, Alzarea AI, Baali FH, et al. Vericiguat Enhances Olfactory Function in Post-Infectious Anosmia: A Randomized Pilot Double-Blind Placebo-Controlled Trial. *Am J Rhinol Allergy* (2025) 39:416–424. doi: 10.1177/19458924251364571

10. Xu C, Scheltema NM, Qi C, Vedder R, Klein LBC, Nibbelke EE, et al. Infant RSV immunoprophylaxis changes nasal epithelial DNA methylation at 6 years of age. *Pediatr Pulmonol* (2021) 56:3822–3831. doi: 10.1002/ppul.25643

11. Damask C, Chen M, Holweg CTJ, Yoo B, Millette LA, Franzese C. Defining the Efficacy of Omalizumab in Nasal Polyposis: A POLYP 1 and POLYP 2 Subgroup Analysis. *Am J Rhinol Allergy* (2022) 36:135–141. doi: 10.1177/19458924211030486

12. Algarni MA, Alharthi MS, Baali FH, Alzarea AI, Alharbi A, Alruqayb WS, et al. Topical Glutamate Diacetate: A Promising Therapy for Post-Coronavirus Disease 2019 Olfactory Dysfunction Through Calcium Modulation. *Am J Rhinol Allergy* (2025) 39:371–378. doi: 10.1177/19458924251347727

13. Chou CT, Valappil B, Mattos JL, Snyderman CH, Gardner PA, Fernandez-Miranda JC, et al. The Effect of Nasoseptal Flap Elevation on Post-Operative Olfaction and Sinonasal Quality of Life: A Prospective Double-Blinded Randomized Controlled Trial. *Am J Rhinol Allergy* (2021) 35:353–360. doi: 10.1177/1945892420957505

14. Puccinelli CL, Yin LX, O’Brien EK, Van Gompel JJ, Choby GW, Van Abel KM, et al. Long‐term olfaction outcomes in transnasal endoscopic skull‐base surgery: a prospective cohort study comparing electrocautery and cold knife upper septal limb incision techniques. *Int Forum Allergy Rhinol* (2019) 9:493–500. doi: 10.1002/alr.22291
